# FDCN-C: A deep learning model based on frequency enhancement, deformable convolution network, and crop module for electroencephalography motor imagery classification

**DOI:** 10.1371/journal.pone.0309706

**Published:** 2024-11-21

**Authors:** Hong-Jie Liang, Ling-Long Li, Guang-Zhong Cao

**Affiliations:** Guangdong Key Laboratory of Electromagnetic Control and Intelligent Robots, College of Mechatronics and Control Engineering, Shenzhen University, Shenzhen, China; Anhui University, CANADA

## Abstract

Motor imagery (MI)-electroencephalography (EEG) decoding plays an important role in brain-computer interface (BCI), which enables motor-disabled patients to communicate with external world via manipulating smart equipment. Currently, deep learning (DL)-based methods are popular for EEG decoding. Whereas the utilization efficiency of EEG features in frequency and temporal domain is not sufficient, which results in poor MI classification performance. To address this issue, an EEG-based MI classification model based on a frequency enhancement module, a deformable convolutional network, and a crop module (FDCN-C) is proposed. Firstly, the frequency enhancement module is innovatively designed to address the issue of extracting frequency information. It utilizes convolution kernels at continuous time scales to extract features across different frequency bands. These features are screened by calculating attention and integrated into the original EEG data. Secondly, for temporal feature extraction, a deformable convolution network is employed to enhance feature extraction capabilities, utilizing offset parameters to modulate the convolution kernel size. In spatial domain, a one-dimensional convolution layer is designed to integrate all channel information. Finally, a dilated convolution is used to form a crop classification module, wherein the diverse receptive fields of the EEG data are computed multiple times. Two public datasets are employed to verify the proposed FDCN-C model, the classification accuracy obtained from the proposed model is greater than that of state-of-the-art methods. The model’s accuracy has improved by 14.01% compared to the baseline model, and the ablation study has confirmed the effectiveness of each module in the model.

## Introduction

Brain-computer interface (BCI) is extensively used in industrial applications, rehabilitative training, and entertainment [1]. BCI converts brain signals into control commands by decoding human motion intention (MI) [2]. Electroencephalography (EEG) is mainly used in the BCI because of its noninvasiveness, high temporal resolution, relative low cost, and high portability [3]. Motor imagery (MI) can generate EEG signals spontaneously. MI-EEG is the common experimental paradigm with event-related synchronization (ERS) and event-related desynchronization (ERD) phenomena [4, 5]. MI-EEG classification is a key component in BCI system to decode and obtain the motor imagery [1]. The main challenge for MI-EEG is how to accurately decode the MI since EEG is non-stationary and low signal-to-noise ratio with various artifact sources [6].

Machine learning (ML) methods have been used for MI-EEG classification, such as common space pattern (CSP) [7] and its improvement variants. The filter bank common spatial pattern (FBCSP) [8] was applied to extract frequency features at different frequency bands, which enhances the ability to use frequency characteristics and improve the accuracy of classification. The classification accuracy of 77% was achieved in the relevant datasets. Miao *et al.* [9] selected the optimal channels and features extracted by CSP and connected them to the feature vector of the sparse representation classification (SRC), the proposed SRC method improved the classification accuracy on the BCI-C III IVa dataset and BCI-C IV 2a dataset by 21.57% and 14.38%, respectively. Sharma *et al.* [10] used a Multi-Layer Perceptron (MLP) and a Support Vector Machine (SVM) to process MI-EEG tasks, achieving a maximum accuracy of 92.5% in binary classification. ML-based methods rely on prior knowledge for EEG feature extraction and require a large number of tuning parameters [11].

Currently, deep learning (DL) methods have been widely applied in MI-EEG. The end-to-end characteristic can reduce the dependence of MI-EEG on specialized knowledge. The typical DL methods have CNN models [12, 13], RNN models [14] and hybrid models [6, 15]. Schirrmeister *et al.* [13] proposed a CNN framework of ShallowConvNet achieved a classification accuracy rate of 72% on the BCI-C IV 2a dataset, and a method based on cropping was designed and improved the model effect. Many researchers referred to the proposed CNN framework for modification and optimization. Lawhern *et al.* [12] proposed a compact CNN model (EEGNet) for EEG-based BCI using deep convolution and separable convolution, it could be applied to MI-EEG classification with support of several EEG paradigms. The classification accuracy of the MI-EEG on the self-built dataset was approximately 75%. Arunabha [16] proposed an efficient multi-scale convolutional neural network (MS-CNN) to extract several typical features of frequency bands, and combined with data augmentation, significantly improved the classification efficiency of EEG, achieving a 93% classification accuracy in binary classification. Zhao *et al.* [17] proposed a multi-branch three-dimensional CNN (MB3DCNN) and a 3D sliding window classification strategy for MI-EEG classification. Ma *et al.* proposed channel-mixing CNN [18], double-branch shallow CNN [19], and multi-branch hybrid neural network [15] to classify MI-EEG tasks. These models utilized CNN to adapt to EEG characteristics of different individual, and channel correlation, temporal feature, spectral features and other EEG features were used to change the model structure, so that a more effective classification model could be constructed. The consideration of adapting specific CNN models to accommodate individual variability in EEG signals, based on features like channel correlations, temporal characteristics, and spectral properties, is an important factor in selecting MI-EEG classification models.

Feature extraction is crucial for MI-EEG classification, due to EEG features are not obvious and intuitional [11]. The classification performance can be improved from the perspectives of the frequency domain, the temporal domain, and the spatial domain [20]. However, feature extraction of EEG data in the frequency domain and the temporal domain remains a challenging process. In the frequency domain, the current module selection includes options such as bandpass filter banks like FBCSP [8], as well as multi-scale convolutional kernels like MS-CNN [16]. Due to the limitations of EEG cognition, it is difficult to divide more suitable frequency bands. In the temporal domain, fixed-length convolution is commonly used, for example, in models like ShallowConvNet [13] and EEGNet [12]. However, the length of temporal features in MI-EEG is not fixed, and using fixed convolutions may limit the effectiveness of extracting longer or shorter temporal characteristics. Addressing this issue requires more flexible methods for extracting temporal features in the time domain. In all, the EEG feature extraction technology needs further improvement.

To address the challenges of feature extraction in the frequency and time domains, this paper proposes an end-to-end MI-EEG classification model. The model is based on a frequency enhancement module, a deformable convolution network, and a crop classification module, named the FDCN-C model. The frequency enhancement module is used for feature processing with three steps: 1) extract features of different frequencies, 2) enhance the features with high-relevance referring to the channel attention mechanism, and 3) add the frequency EEG features to the raw data. The convolution kernels cover a broader frequency range, making the extracted data easier to decode. Secondly, in temporal domain, a deformable convolutional network (DCN) with trainable parameter offsets is designed to control the deformation and motion of the convolution kernel to fully extract the temporal features. In spatial domain, a one-dimensional convolution is used to fuse EEG features of different channels. Finally, a one-dimensional dilated convolution is adopted for multiple calculations to find the mean value for MI-EEG classification, called the crop classification module. The prediction average value in different receptive fields is calculated to improve the classification accuracy. Two public datasets, the BCI Competition IV 2A dataset [21] and the High Gamma Dataset [13] are used to validate the proposed FDCN-C model, which yields better results than other comparison methods.

Main contributions of this paper are summarized as follows:

1. A frequency enhancement module based on the continuous-scale convolution kernel is proposed. The convolution kernel of the continuous time scale is firstly used to extract features of different domains, and the channel attention mechanism is used to select effective features. Finally, different frequency features are fused in the original EEG data.
2. A temporal dimension feature extraction module based on DCN is proposed. The DCN is used to replace the fixed-length convolution or multiple parallel fixed-length convolutions and enhance the ability of extracting EEG features for the convolution network.
3. The dilated convolution is designed to compose the crop classification module. The classification accuracy is improved by calculating the average prediction value across different receptive fields.

The remainder of the paper is organized as follows. Section 2 introduces the structure of the proposed FDCN-C model. Section 3 addresses the experimental process for validation. Section 4 analyzes the FDCN-C model and discusses the experimental results. Section 5 draws the conclusion of this paper.

## Proposed model

**Fig 1:**
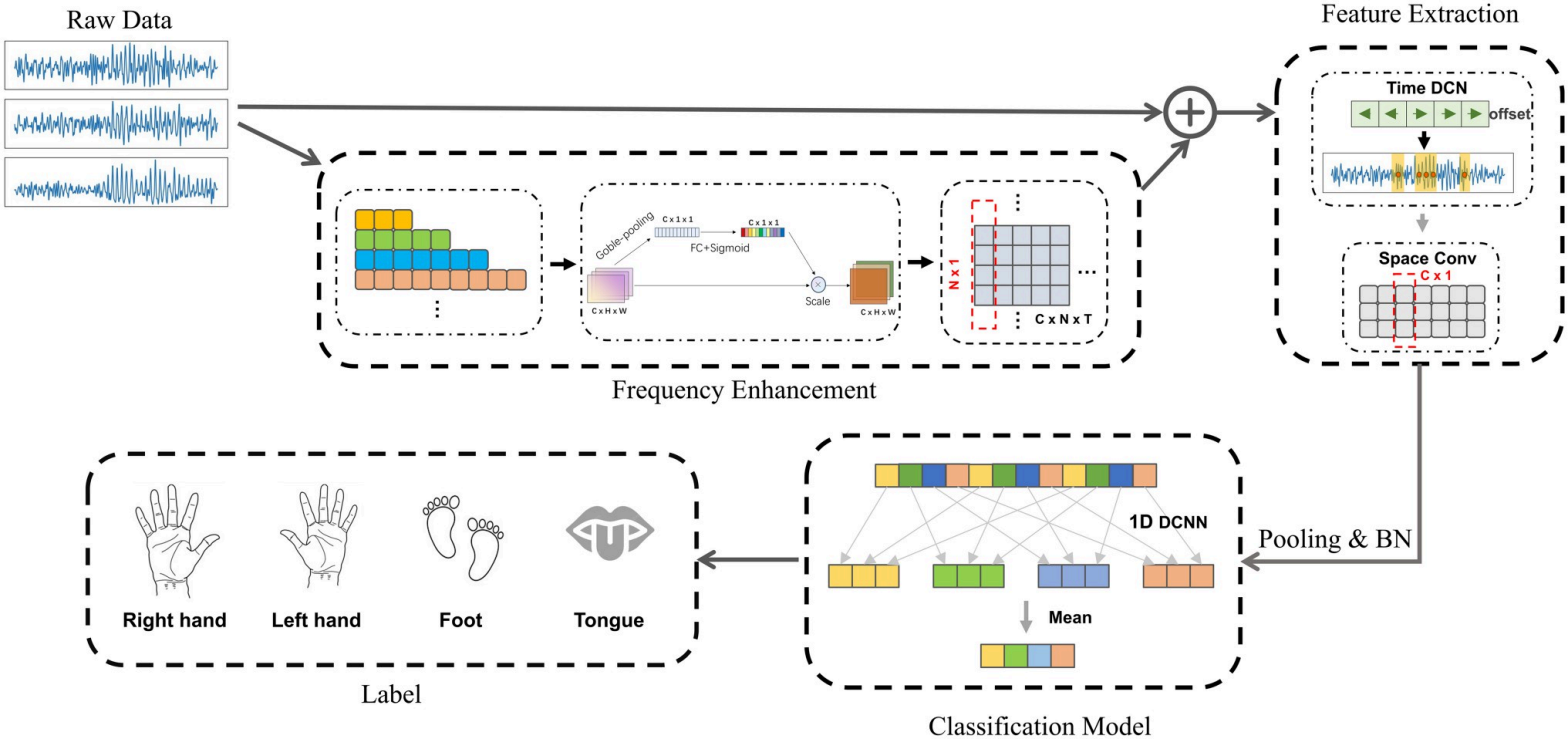
The overall structure of FDCN-C model. The FDCN-C model has a frequency enhancement module, a feature extraction module, and a classification module, *C* is the number of EEG channels, *N* is the number of convolution of frequency enhancement modules, *T* represents the temporal length of the EEG signal.

The proposed FDCN-C model consists of a frequency enhancement module, a feature extraction module, and a classification module. The overall structure of the FDCN-C model based on CNN architecture is shown in Fig 1.

The FDNC-C model refers to the mainstream CNN of ShallowConvNet [13], EEGNet [12], and EEG-TCNet [22]. The input is raw EEG data, and EEG features of temporal and spatial domains are extracted. Then the extracted features are deeply processed, and the classification module outputs the classification class representing the MI-EEG task.

The frequency enhancement module involves an extraction module with continuous time scale convolutions, a channel attention mechanism module for weight calculation, and an information integration module. The feature extraction module extract features in temporal dimension and spatial dimension. The classification module is composed of one-dimensional extended convolution, and the prediction values of different receptive fields of EEG features are calculated.

### Feature enhancement module

#### Continuous time scales convolution kernel

To utilize the frequency information in EEG data, various transforms like Fourier transform [23], windowed Fourier transform [24], and wavelet transform [25] have been applied to generate spectrum diagrams as inputs, although these methods can lead to information loss and have distinct advantages and disadvantages. Neural networks are capable of extracting frequency domain features from EEG data. Xin *et al.* [26] used wavelet CNN based on the attention mechanism to extract frequency dimension features without generating spectral graphs.

The basic principle of this module design is to create an adaptive feature extraction module in the frequency domain of EEG processing, which is more capable of accommodating the uncertain distribution of effective frequencies in MI-EEG compared to existing methods. The convolution kernels of different lengths are introduced to extract frequency features in the proposed model. Similar to the wavelet transform, the wavelet basis functions with different length are used to calculate the correlation in transformation.

The length of the convolution kernel can be calculated as

$$\begin{array}{c} \begin{array}{cc} & M_{\text{max}}=\frac{f_{s}}{f_{\text{max}}}\\ & M_{\text{min}}=\frac{f_{s}}{f_{\text{min}}}, \end{array} \end{array}$$ (1)

where *f*_max_ and *f*_min_ are the upper and lower limits of EEG frequency range, *f*_*s*_ is the sampling frequency, *M*_max_ and *M*_min_ are the upper and lower limits of convolution kernels size.

Using multiple convolution kernels of varying lengths allows coverage of a wider frequency range, enhancing the extraction of valuable data compared to traditional methods like wavelet transforms. This approach provides a more comprehensive analysis of the signal, capturing a broader spectrum of features that are crucial for accurate classification. Referring to the [2], the frequency range is set as 4-38Hz, the number of convolutional kernels is set to 131. Padding parameters are appropriately set to ensure the input and output shapes are congruent.

#### Channel attention module

The channel attention mechanism was once used to enhance the performance of image classification, by calculating different channels of the feature map [27]. Y. Li *et al.* [28], He *et al.* [29], and D. Li *et al.* [30] adopted the channel attention mechanism in their proposed models, and verified the validity of their models. The reason for selecting this module is to enhance the representation effectiveness of each frequency component obtained in the previous module. It is based on a squeeze-and-excitation (SE) block [27]. The squeeze operation leverages subsequent transformations to embed global information and compresses the information into channel descriptors by the way of global average pooling. The excitation operation adopts the gating mechanism of two full connection (FC) layers and uses sigmoid as the activation function. The obtained sequence through squeeze and excitation calculation is inter-channel attention, which can be regarded as the attention for the EEG features of different frequency bands. The output value of channel attention is multiplied by the input data of different channels. So that the effective EEG features are augmented and invalid features are suppressed. The SE module is an effective strategy for feature selection, which guides the network to focus on the salient parts [27]. In the frequency enhancement module, the SE module is employed to compute attention across different frequency bands. This process increases the weight proportion of information from effective frequency ranges, thereby preserving more relevant information.

#### Integration module

A one-dimensional convolution kernel is used to integrate all extracted features in reference with the weights and superimpose the raw EEG data. The total process of the frequency enhancement module is a linear transformation of the EEG data.

#### Calculation method

The calculation method of the frequency enhancement module is

$$\begin{array}{c} Out(1,p,k)=input(1,p,k)+\sum_{j=0}^{C-1}W_{fm}\cdot \{W_{se}(j)\cdot [\sum_{i=0}^{C_{f}-1}W_{fe}\cdot input(1,p,k)]\}, \end{array}$$ (2)

where *W*_*fe*_ represents the weight of the convolution kernel during frequency feature extraction, $\sum_{i=0}^{C_{f}-1}\;W_{fe}\cdot input(N,k)$ represents the calculation of continuous timescales convolution, *C*_*f*_ is the number of continuous time scales convolution kernels, *input*(1, *p*, *k*) represents the raw EEG data with the shape of (*N* × *T*). *W*_*se*_ is the channel weight obtained by channel attention mechanism, the expression *W*_*se*_ ⋅ *A* represents the addition of attention weights to the extracted features across different frequencies, *W*_*fm*_ represents the weight of frequency feature integration, and $\sum_{j=0}^{C-1}W_{fm}\cdot A$ represents the calculation of integration module. *p*, *k* is the index of the location of the data.

In Eq (2), that data is extracted by continuous convolution kernels, the weight is added by SE block, then integration is carried out, and the data is superimposed with the original data.

### Feature extraction module

#### Temporal domain extraction

Convolution of adaptive transformations has been proposed to feature extraction. Dai *et al.* [31] designed a deformable convolution based on augmenting the spatial sampling position with extra offsets and learning the offsets from the target task without additional supervision. Zhu *et al.* [32] improved Dai *et al.*’s [31] approach by adding a learned feature amplitude to focus on relevant image regions. Then deformable convolution was transferred from visual task to temporal recognition task. Shen *et al.* [33] proposed a vehicle speed prediction method based on temporal cluster analysis and deformable convolutional neural networks (DCNs), demonstrating superior performance in vehicle speed prediction. Bhagya *et al.* [34] developed a 1D deformable CNN to diagnose chronic obstructive pulmonary disease and congestive heart failure through analyzing capnograms. Xu *et al.* [35] proposed a deformable CNN for human activity recognition from complex sensory data, variable convolution was used to replace the convolutional layer and achieved significant improvement in multiple wearable sensor human activity recognition datasets.

The principle behind selecting the time-domain feature extraction module is its ability to adapt to the varying lengths of temporal features in MI-EEG. Compared to fixed convolutional kernels, DCNs can enhance network sensitivity and improve the ability to extract effective features. DCNs are particularly advantageous in handling non-stationary and low signal-to-noise ratio data typical in EEG signals. This adaptability allows the network to better capture and interpret the subtle and variable patterns within EEG data, leading to more accurate and robust classification performance.

**Fig 2:**
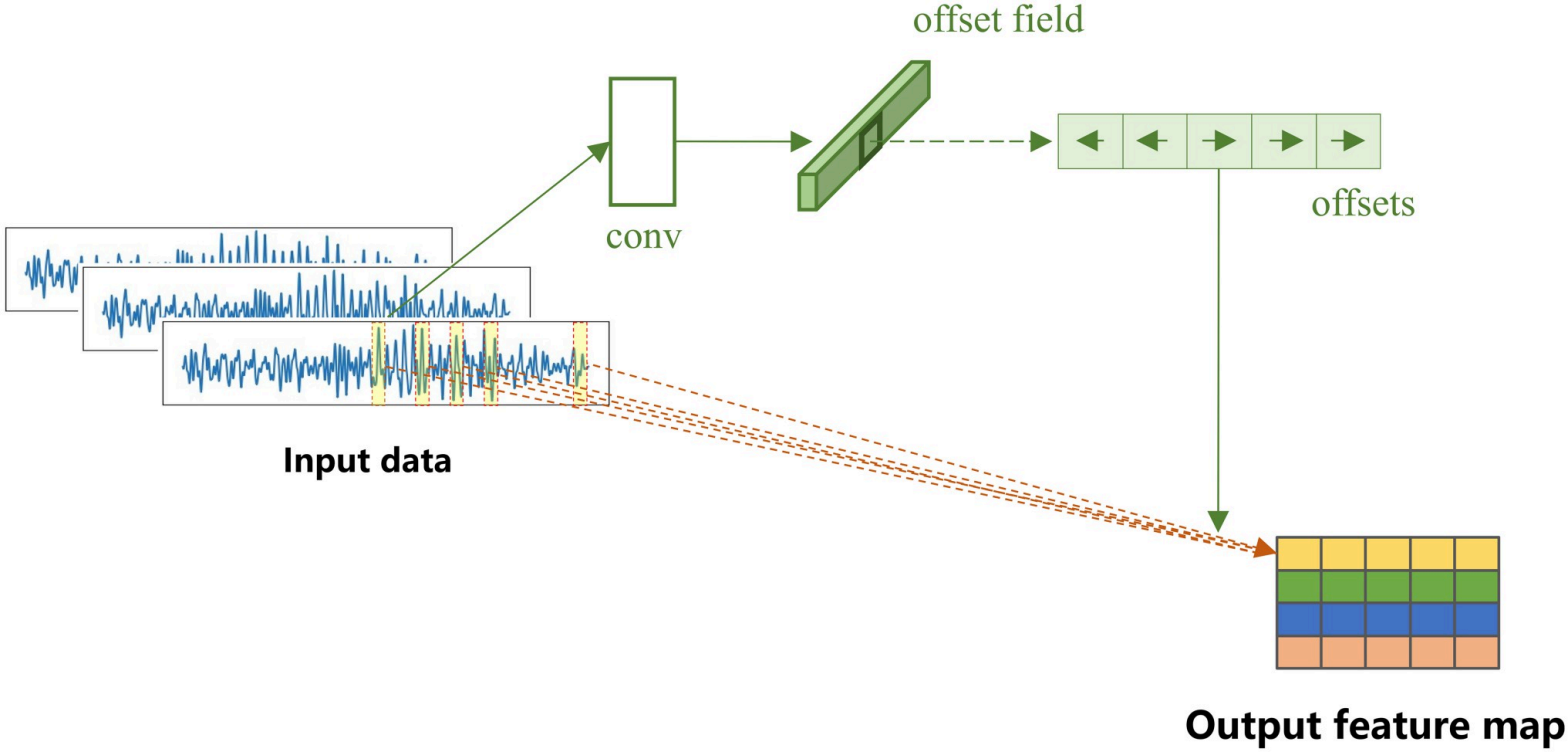
Deformable convolution network (DCN).

Therefore, we attempt to apply DCN in MI-EEG tasks. The deformable convolution kernel is designed by referring to deformable convnets v2 [32], the structure of the DCN model is illustrated in Fig 2. Empirical evidence and theoretical explanations support the superiority of DCNs over standard convolutional networks. By allowing the convolutional sampling grid to adapt to the most informative parts of the input data, DCNs can significantly improve the extraction of relevant features, leading to enhanced performance in EEG signal classification tasks.

As shown in the Fig 2, the two key differences between DCN and traditional convolution are: (1) generating offsets and (2) generating deformable convolution kernels. The offsets are obtained by applying a convolutional layer on the same input features. The convolutional layer has a kernel size of 1 × *n*, where *n* represents the extracted time length, which is set to 15 in this paper. The output offset fields have the same spatial resolution with the input feature map. The channel dimension *n* corresponds to the size of 1D offsets. During the training process, both the generation of the output features’ convolution kernels and offsets are learned simultaneously.

The convolution operation formula is

$$\begin{array}{c} Out(c,p,k)=\sum_{i=0}^{C-1}W_{i}\cdot input(1,p,k+k_{i}+\Delta k_{i})\cdot \Delta m_{i}, \end{array}$$ (3)

where *p*, *k* is the index of the location of the feature, the input has a dimension of 1, whereas the output has a dimension of *c*. Δ*k*_*i*_ and Δ*m*_*i*_ are the learnable offsets and modulation scalar for the k-th location, *C* is the number of convolution kernel. The modulation scalar Δ*m*_*i*_ lies in the range [0, 1], while Δ*k*_*i*_ is a real number without constraint. Bilinear interpolation is applied in [31] in computing offset position. Both Δ*k*_*i*_ and Δ*m*_*i*_ are obtained by a separate convolution layer applied over the same input feature maps. The initial kernel weights in this separate convolution layer are 0. The initial values of Δ*k*_*i*_ and Δ*m*_*i*_ are 0 and 0.5, respectively. The learning rates of the added convolution layers for offsets and modulation are 0.1 times the existing layers.

#### Spatial domain integration

After the EEG data is processed by the previous module, the data form *N* dimensions in space, where *N* is the electrode number of the input. Schirrmeister *et al.* [13] and Lawhyn *et al.* [12] used a one-dimensional convolution to extract features in spatial domain, in order to get the common information across channels. This paper uses a one-dimensional convolution to extract EEG features in temporal domain. Referring to [36], we integrate the same effective information and use convolution calculation to compress all features in the spatial dimension. The formula of the one-dimensional convolution is

$$\begin{array}{c} Out(c,1,k)=\sum_{k=0}^{K-1}W_{c}\cdot input(c,p,k), \end{array}$$ (4)

where the size of the convolution kernel is *p*, the output shape of spatial domain is 1.

The use of one-dimensional convolution to integrate EEG features across channels is advantageous because it efficiently compresses spatial information while retaining crucial temporal features. This approach captures the temporal dependencies of EEG signals, essential for accurate classification and interpretation. Compared to two-dimensional convolutions or traditional methods [16], it is more computationally efficient, better captures the dynamic nature of EEG signals, and enhances model robustness and generalization across different datasets. In summary, integrating one-dimensional convolution for temporal feature extraction with spatial dimension compression improves model performance by effectively capturing temporal dynamics and reducing computational complexity.

### Classification module

The principle of the classification process in the cropping module is similar to that of using segmented data, but it achieves better results and is more suited to MI-EEG tasks [13]. In general, the output of the classification module is derived directly from the flatten layer and the fully connected network [17, 18]. Several researchers refer to FCNN model [37] and use convolution methods to reduce the number of parameters. And the dilated convolution methods are employed to increase the performance of the network, such as Giusti *et al.* [38] and Long *et al.* [37]. Schirrmeister *et al.* [13] applied the dilated convolution to the classification layer of MI-EEG decoding and called the crop module. During the classification process, the classification model accepts EEG data from various receptive fields. Multiple calculations are subsequently conducted to enhance the fault tolerance of the classification model. The dilated convolution and average value calculation are used to calculate the different sensitive fields of EEG data several times, to improve the classification accuracy. The reason for selecting the crop module is its capability to improve the training efficiency of the model at a minimal increase in inference computations. The calculation method of this module is expressed below

$$\begin{array}{c} Out(p)=\frac{\sum_{m=0}^{M-1}[\sum_{k=0}^{K-1}w_{k}\cdot input(x+dx)]}{M}, \end{array}$$ (5)

where *d*_*x*_ represents the dilated coefficient of the dilated convolution, *K* is the number of convolution kernels whose value is equal to the class of the classification, and *M* means the output vector dimension of the convolutional layer. Specifically, the stride of the convolutional layer and pooling layer in the above module is set to one, and the dilated coefficient of the classification model equals the product of the initial stride in all the previous layers. The dilated convolution output is a sequence, and the maximum mean value is used to determine the final output class.

### Loss function

The loss function of the model is cross-entropy, and the calculation formula is

$$\begin{array}{c} L=\frac{1}{N}\sum_{i}\sum_{c=1}^{N}\;y_{ic}\;\text{log}(p_{ic}), \end{array}$$ (6)

where *y* is a symbolic function, representing the class of *i*, *p* is the output probability, *N* is the number of classes in the labels.

## Experiment setup

To verify the reliability of the FDCN-C model, we adopt experiments on two public datasets, the BCI Competition IV 2A dataset [21] and the High Gamma Dataset [13].

### Dataset

#### BCI competition IV 2A dataset

The BCI Competition IV 2A (BCI-C IV 2a) dataset consists of EEG recordings from 9 subjects using 22 electrodes. There are four visualization MI-EEG tasks, imagining movements of the left hand (class 1), right hand (class 2), foot (class 3), and tongue (class 4). Each subject has two sessions, and there are 288 trials in each session. The former session is used as the training set, and the latter session is used as the test set.

#### High gamma dataset

The High Gamma Dataset (HGD) contains EEG recordings of 14 subjects with 44 electrodes related to the motor cortex. Each subject completed four MI-EEG tasks: left hand (class 1), right hand (class 2), resting (class 3), and feet (class 4). The original sampling frequency was 500Hz. In this experiment, it is downsampled to 250Hz. There are 880 training set samples and 160 test set samples for each subject.

### Data preprocessing

The EEG data reading adopts the MNE [39] library in Python, with data and labels taken directly from the original dataset without modification during the data reading phase. The preprocessing steps include data segmentation, low-pass filtering, and data standardization, each enhancing signal quality and classification performance. Data segmentation divides continuous EEG signals into epochs, allowing for temporal localization and reducing complexity. Low-pass filtering removes high-frequency noise, improving the signal-to-noise ratio and ensuring that relevant low-frequency components are preserved. Data standardization scales the EEG data to have a mean of zero and a standard deviation of one, normalizing the features so that all contribute equally to the model’s learning process and improving convergence during training. These preprocessing steps collectively ensure a robust and accurate classification model, capable of effectively interpreting and analyzing EEG data.

#### Low-pass filtering

The 8-order Butterworth low-pass filter with an upper frequency of 38Hz is used to eliminate electromagnetic interference and artifact produced by electrocardiogram (ECG) and electromyography (EMG) while retaining the key features of EEG [20].

#### Data standardization

Data standardization aims to keep the EEG data ranging in [−1, 1]. The standardization is following (7), and implemented on both the training and test sets.

$$\begin{array}{c} {\tilde{X}}_{i}=\frac{1}{Var}\cdot (X_{i}-E), \end{array}$$ (7)

where $\tilde{X}$ is the preprocessed output data, *i* is the point of the data, *E* and *Var* stands for the mean and the variance of the training data sets in one session.

### Data augmentation

Due to the inconvenience of EEG acquisition, EEG datasets are typically small in scale, which can lead to overfitting in deep learning (DL)-based models. To mitigate this, data enhancement methods are crucial and can be categorized into frequency domain methods, temporal domain methods, and spatial domain methods [40]. This paper employs data enhancement techniques from all three perspectives to improve model performance and generalization.

In the frequency domain, a Fourier transform surrogate is used where the original EEG signal undergoes a fast Fourier transform (FFT). The phase of the Fourier coefficients is then randomized while keeping the amplitude constant by adding random noise to the phase component. The signal is then transformed back to the time domain using an inverse FFT, resulting in a new signal with the same amplitude spectrum but a randomized phase. In the temporal domain, Gaussian noise is added to the EEG signals, which helps the model become more robust to slight variations and noise. Additionally, a signal smooth mask is used to cover part of the time information, encouraging the model to focus on critical parts of the signal and preventing overfitting to specific temporal patterns.

These data augmentation methods increase variability in the training data, improve robustness to noise, and enhance generalization to unseen data. Consequently, they significantly enhance the robustness and generalization capability of DL-based models for EEG signal classification, ensuring that the models perform well on new, unseen data.

### Training implementation

The experiments are conducted on Windows 10 64-bit system with NVIDIA RTX 3060 GPU for training and validating the proposed model. The experiments were conducted using Python 3.10, utilizing the mne library version 0.24.1 for reading the raw EEG signals. The model architecture was constructed using PyTorch 1.12.0. The number of training epochs was set to 100, the batch size was configured as 64. The Adam optimization algorithm is used to minimize the loss function using a constant of 6.25e-4 as the learning rate of the network.

The model parameters of FDCN-C model depicted in Table 1, which *N* is the electrode number of the input.

**Table 1:** The network parameters of the FDCN-C model.

Modules	Filter Numbers	Kernel Sizes
Frequency domain extraction	131	–
Frequency domain integration	*N*	(131,1)
Temporal domain extraction	40	(15,1)
Spatial domain extraction	40	(1, *N*)
Pooling	–	(15,1)
Classification module	4	(20,1)

The FDCN-C model with the parameters from Table 1 has a total of 196.57M floating-point operations (flops) and 0.14M parameters. Compared to the baseline model (ShallowConvNet [13]), it exhibits an increase of 33.17% in flops (compared to 147.61M flops) and a 40% increase in parameters (compared to 0.10M parameters) However, it still has lower FLOPs and parameters than the DeepConvNet [13], which has 252.05 million flops and 0.35 million parameters.

### Evaluation metric

#### Accuracy

Accuracy is one of the indexes used to evaluate the classification performance of the proposed model. The classification accuracy *acc* is calculated as shown in

$$\begin{array}{c} acc=\frac{TP+TN}{TP+TN+FP+FN}\;acc\in R[0,1], \end{array}$$ (8)

where *TP*, *TN*, *FP* and *FN* represent true positives, true negatives, false positives, and false negatives, respectively. Accuracy measures the proportion of correctly classified instances out of the total instances. The higher the accuracy, the better the model’s classification performance.

#### Hypothesis Test

The hypothesis test is conducted to determine whether the conclusion obtained by the FDCN-C model is superior to the comparative models with statistical significance. Specifically, the hypothesis is that there is no significant difference between FDCN-C and one of the comparison models, and the corresponding data is selected for Student’s t-test [41] to calculate the *P*-value. The *P*-value is a measure of the probability that the observed differences between models occurred by chance. A lower *P*-value suggests that the observed difference is less likely to be due to random variation, thereby providing stronger evidence that the FDCN-C model performs differently compared to the comparison model. This statistical validation is crucial for establishing the reliability and robustness of the model’s performance.

## Experiment results and discussion

### Comparison models

To verify the effectiveness of the FDCN-C model, we compare it with the the state-of-the-art (SOTA) models, which are introduced as follows.

1. ShallowConvNet: It uses a 3-layer CNN model and a pooling layer to decode EEG signals, its nonlinear part refers to FBCSP [13].
2. DeepConvNet: The CNN with tens of layers to decode EEG signals, the nonlinear module uses ELU [13].
3. EEGNetv4: Starting from temporal and spatial convolution, and then separable convolution, based on depth convolution, which can reduce the time of convolution operation [12].
4. EEGITNet: An end-to-end deep learning architecture proposed by Salami *et al.* Using inception modules and causal convolutions extract spectral, spatial, and temporal information from multi-channel EEG signals [42].
5. EEGInceptionMI: Using several inceptions and residual modules as its backbone, proposed by Zhang *et al.* [43], effectively enhancing performance in a lightweight architecture
6. ACTNet: Proposed by Altaheri *et al.* [44], employed multihead self-attention to highlight the most valuable features in MI-EEG data, temporal convolutional network to extract high-level temporal features.
7. FBCNet: Proposed by Mane *et al*. [45], combines neuro-physiological inspiration, filter-bank convolutional network architecture, multi-view data representation, and a Variance layer. It extracts spectro-spatially discriminative features through spatial filtering and efficiently trains with limited data.

In this paper, the ShallowConvNet serves as the baseline model, while the BCI-C IV 2a Dataset and HGD are the benchmark datasets. To facilitate a fair comparison, we retrained the models employed in the published journal using identical test set partitioning, data augmentation methods, and standardization techniques.

### Results on the BCI-C IV 2a Dataset

**Fig 3:**
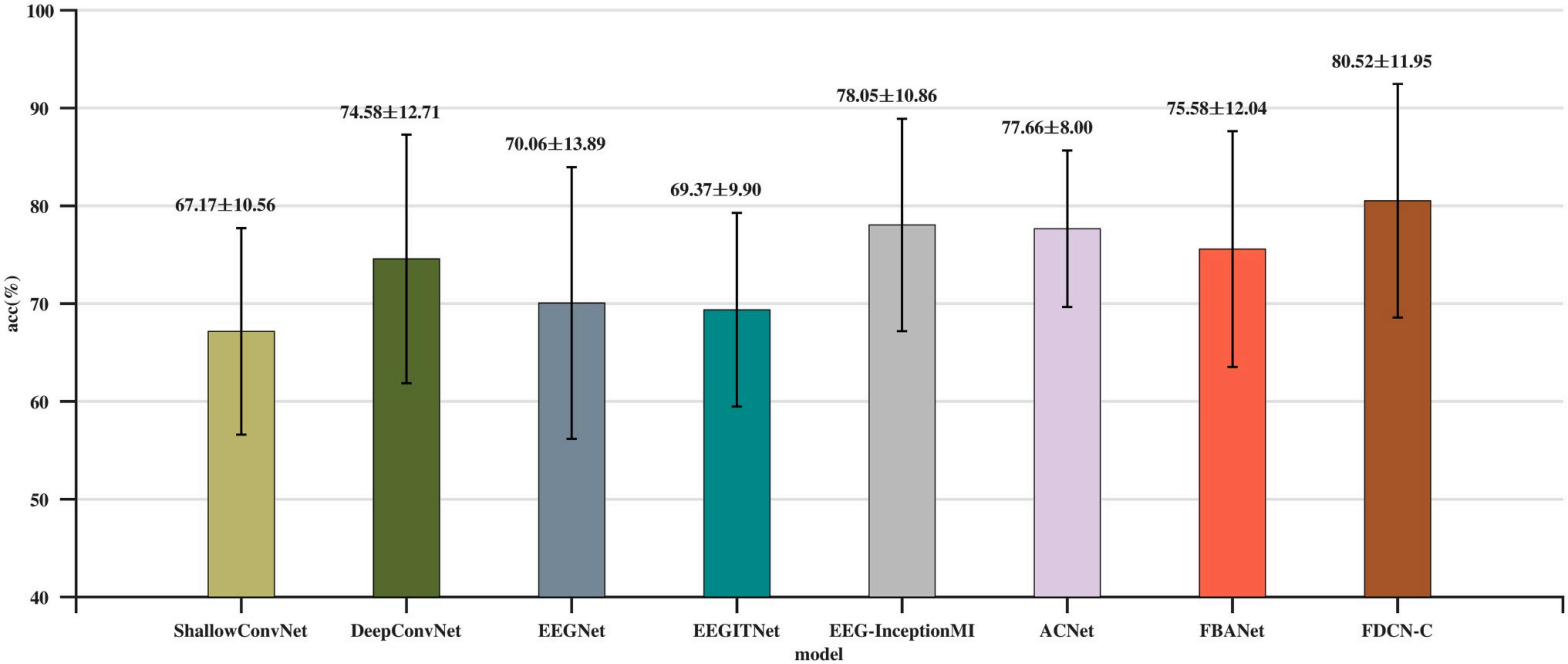
Comparison of average classification accuracy (%) and standard deviation on the BCI-C IV 2a dataset.

Table 2 shows the classification result of the proposed method and other comparison methods, the character in bold represents the highest classification accuracy in subjects, the *P*-value represents the significance analysis of the model’s data compared to the FDCN-C model. Fig 3 depicts the comparison results.

**Table 2:** Comparison of classification accuracy (%) on the BCI-C IV 2a dataset.

	ShallowConvNet	DeepConvNet	EEGNet v4	EEGITNet	EEGInceptionMI	ACTNet	FBCNet	Proposed FDCN-C
**A1**	68.40	79.86	77.78	78.13	86.11	78.47	84.72	**89.24**
**A2**	50.35	49.65	51.04	48.61	56.60	**62.15**	60.07	59.38
**A3**	84.72	86.81	90.28	80.21	92.71	90.28	90.28	**93.75**
**A4**	67.36	66.32	60.42	64.93	76.74	76.39	73.96	**77.43**
**A5**	66.32	71.18	56.25	68.40	70.83	**77.43**	62.15	70.14
**A6**	49.65	60.76	53.13	59.03	67.01	**67.36**	57.64	**67.36**
**A7**	71.53	87.85	81.25	70.83	87.50	83.68	89.24	**95.14**
**A8**	70.49	87.15	81.60	75.00	85.42	82.29	78.47	**89.24**
**A9**	75.69	81.60	78.82	79.17	79.51	80.90	**83.68**	82.99
**avg**	67.17	74.58	70.06	69.37	78.05	77.66	75.58	**80.52**
**std**	10.01	12.05	13.17	9.39	10.30	7.59	11.43	11.33
***P*-value**	0.000428	0.002784	0.000176	0.000900	0.017884	0.1997384	0.00691	———

Based on the data in the Table 2, FDCN-C demonstrates good performance across different metrics. It achieves higher accuracy than other models in A1, A3, A7, and A8, and performs better than most models in the majority of the metrics. This indicates that FDCN-C is likely an effective choice for the evaluation task, with high accuracy and performance. From the *P*-values listed in Table 2, ShallowConvNet, DeepConvNet, EEGNet v4, EEGITNet, EEGInceptionMI, and FBCNet show statistically significant differences compared to FDCN-C.

### Results on the HGD

**Fig 4:**
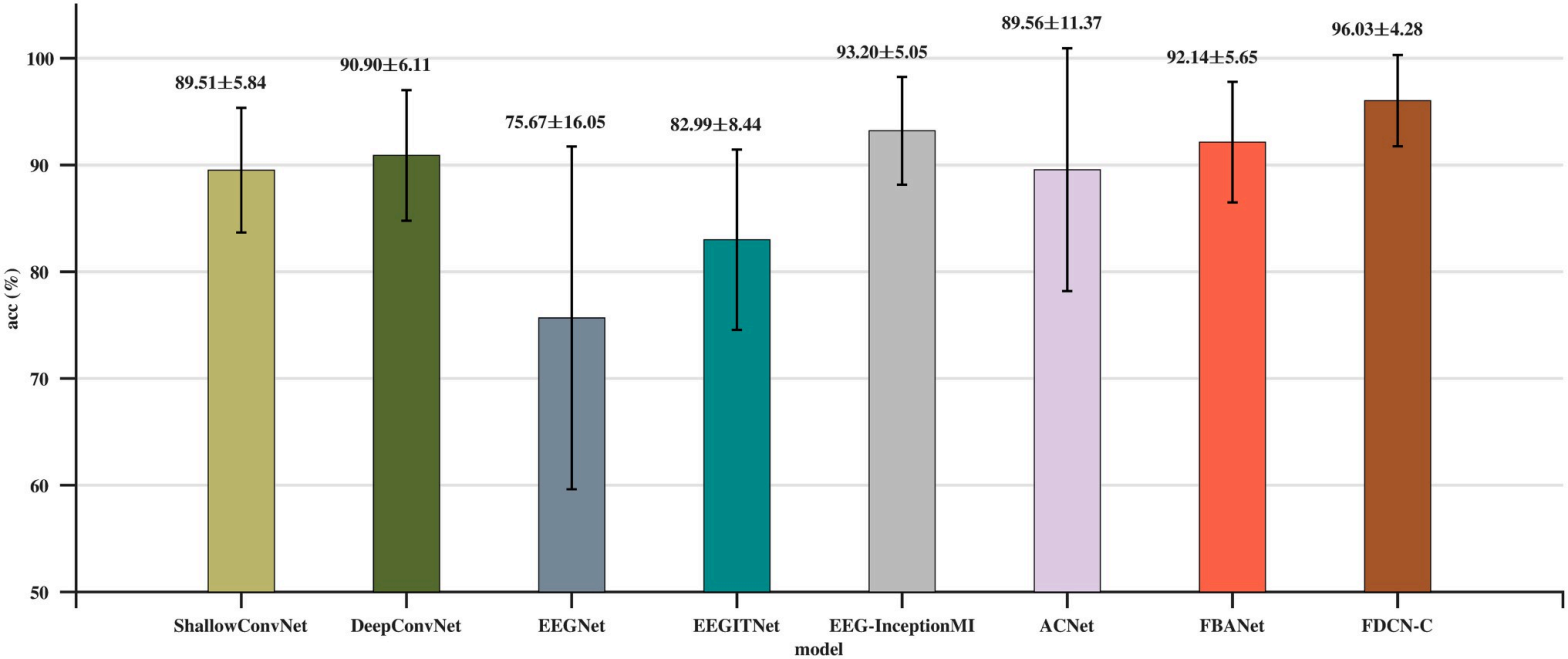
Comparison of average classification accuracy (%) and standard deviation on the HGD.

As shown in Table 3 and Fig 4, the average classification accuracy of FDCN-C is 96.30%, which is better than that obtained by ShallowConvNet, DeepConvNet, EEGNet v4, ACTNet, and FBCNet. In terms of variance, the FDCN-C model exhibits a accuracy variance of 4.13, which is the lowest among the compared methods, indicating that the model has a stable classification performance across different subjects. The statistical analysis reveals that FDCN-C outperforms other models significantly, with *P*-values well below the threshold of 0.05.

**Table 3:** Comparison of classification accuracy (%) on the HGD.

	ShallowConvNet	DeepConvNet	EEGNet v4	EEGITNet	EEGInceptionMI	ACTNet	FBCNet	Proposed FDCN-C
**S1**	91.25	83.13	81.25	90.00	95.13	94.38	86.88	95.00
**S2**	85.63	83.75	63.125	76.25	95.75	82.50	91.25	95.63
**S3**	96.88	98.13	83.75	91.25	98.13	98.13	99.38	98.75
**S4**	93.13	96.88	92.50	90.00	98.75	98.75	98.75	100.00
**S5**	94.38	96.88	86.88	90.00	95.00	95.63	95.00	98.13
**S6**	88.75	90.63	66.25	78.13	93.75	93.13	93.13	100.00
**S7**	76.88	85.63	41.88	69.38	90.63	56.88	86.25	91.88
**S8**	91.25	92.50	84.38	84.38	89.38	91.88	97.50	98.75
**S9**	90.63	98.75	93.75	93.13	97.50	96.88	82.50	100.00
**S10**	89.38	93.75	90.00	86.25	92.00	92.50	95.00	95.63
**S11**	77.50	82.50	48.13	67.50	80.63	72.50	81.25	83.75
**S12**	96.25	94.38	86.88	92.50	98.75	96.25	97.50	98.75
**S13**	93.13	94.38	79.38	76.88	93.75	96.88	93.13	95.63
**S14**	88.13	81.25	61.25	76.25	85.63	87.50	92.50	92.50
**avg**	89.51	90.89	75.67	82.99	93.20	89.55	92.14	96.30
**std**	5.65	5.91	15.51	8.16	4.88	10.98	5.46	4.13
***P*-value**	0.000024	0.000682	0.000089	0.000002	0.002809	0.020323	0.008550	———

### Analysis of ablation study

**Fig 5:**
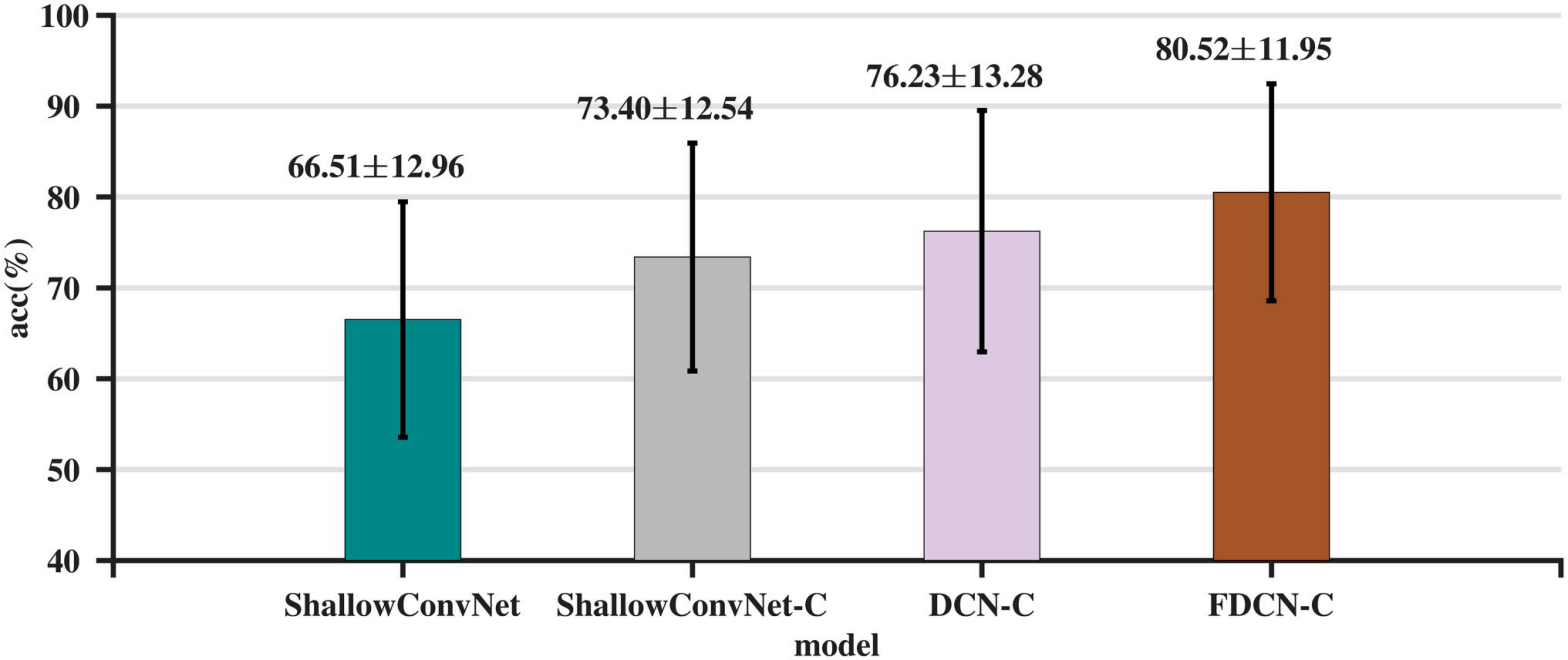
Average classification accuracy (%) and standard deviation of ablation study on the BCI-C IV 2a dataset.

To verify the effectiveness of each module in the FDCN-C model, an ablation study is conducted on the BCI-C IV 2a dataset. Comparisons are made using ShallowConvNet, ShallowConvNet with crop module (ShallowConvNet-C), deformable convolution network with crop module (DCN-C), and frequency enhancement DCN-C (FDCN-C). Results of the ablation study are presented in Table 4 and Fig 5. The FDCN-C model demonstrates superior average classification accuracy in comparison to other models, exhibiting respective increases of 14.01%, 7.12%, and 4.28%. Statistical hypothesis tests were conducted between FDCN-C and ablation models, all *P*-values were found to be less than 0.05, indicating a statistically significant difference between FDCN-C and its ablation models. These results demonstrate the effectiveness of the frequency enhancement module, DCN, and crop module in enhancing the decoding ability.

**Table 4:** Comparison of classification accuracy (%) on the BCI-C IV 2a dataset.

	ShallowConvNet	ShallowConvNet-C	DCN-C	Proposed FDCN-C
**A1**	83.68	84.45	86.11	**89.24**
**A2**	49.31	50.60	53.13	**59.38**
**A3**	83.33	89.58	92.01	**93.75**
**A4**	61.46	75.43	**77.78**	77.43
**A5**	54.17	63.89	65.97	**70.14**
**A6**	49.65	56.64	57.99	**67.36**
**A7**	72.92	82.24	90.97	**95.14**
**A8**	65.97	78.99	82.29	**89.24**
**A9**	78.13	78.47	79.86	**82.99**
**avg**	66.51	73.40	76.23	**80.52**
**std**	12.96	11.90	12.60	**11.33**
***P*-value**	0.000238	0.000425	0.002220	———

The effectiveness of the crop module can be observed from Table 4 and Fig 5, where the performance of the ShallowConvNet-C module surpasses that of the baseline model ShallowConvNet. The crop module improves the model’s accuracy and robustness by focusing on the most relevant portions of the input data, effectively reducing noise and irrelevant information. The results also indicate that, in the process of extracting temporal features, the deformable convolution module in the DCN-C model can extract more effective features compared to the fixed-length convolution module in the ShallowConvNet-C model. In the comparison results between the FDCN-C model and the DCN-C model, the frequency enhancement module improves the DCN-C model’s ability to extract features in the frequency domain, thereby enhancing the performance of the model. Overall, the FDCN-C model achieves the highest classification performance among the tested models.

### Analysis of visualization

#### Analysis of T-SNE

**Fig 6:**
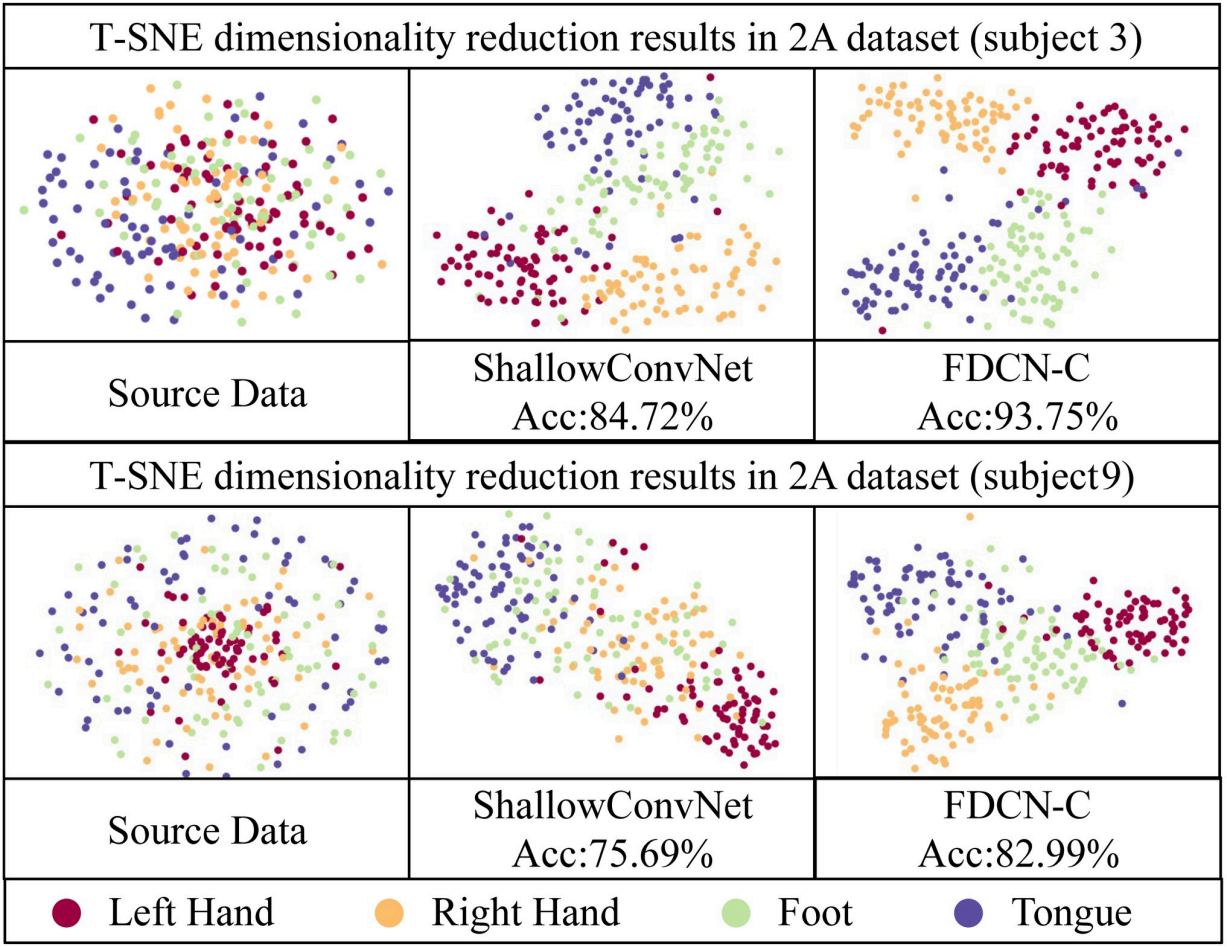
T-SNE result in the BCI-C IV 2a dataset. The selected subjects of the BCI-C IV 2a dataset are subject 3 and subject 7.

**Fig 7:**
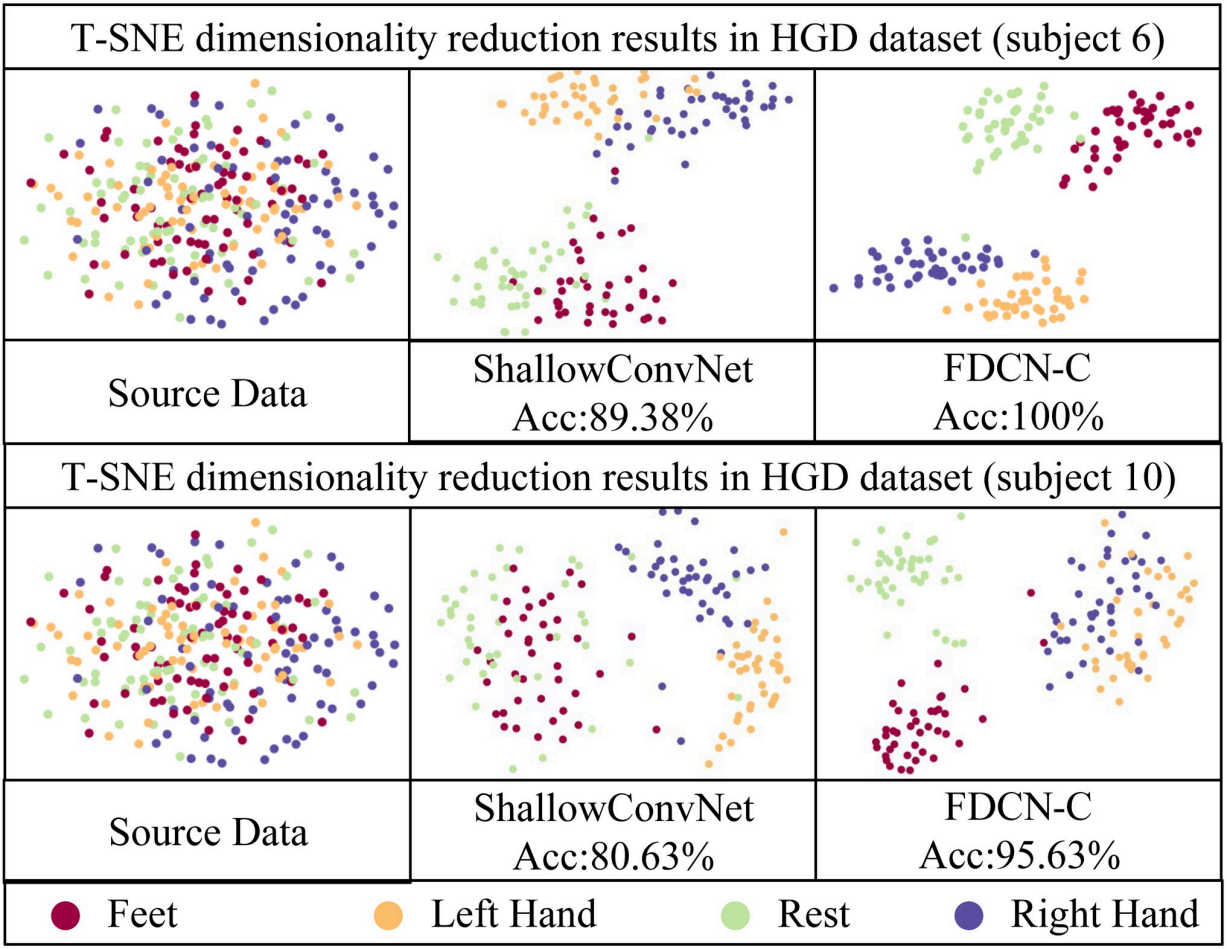
T-SNE result in the HGD. The selected subjects of the HGD dataset are subject 6 and subject 10.

To study the distinguishability of the features extracted by FDCN-C in detail, T-SNE (t-Distributed Stochastic Neighbor Embedding) [46] was used to visualize the learned features. T-SNE was employed to reduce the feature vectors output from different model feature extraction layers into two-dimensional data. Subsequently, these reduced-dimensional data points were plotted on Figs 6 and 7. The model for comparison is the baseline model ShallowConvNet. The comparison data were selected for subjects 3 and 7 in BCI-C IV 2a dataset and subjects 6 and 10 in HGD.

Figs 6 and 7 shows that the feature extraction module of FDCN-C performs the correct function, which is able to distinguish the classes of MI-EEG tasks from EEG features obviously on the BCI-C IV 2a and HGD. The distinctive EEG features are extracted effectively.

As can be seen from the Figs 6 and 7, compared with ShallowConvNet, the 2D images visualized in FDCN-C show more obvious distinctions between different classes, the same class is more clustered, and the 2D image boundaries of ShallowConvNet are more blurred. This confirms that FDCN-C captures a greater number of discernible features from raw EEG signals. Frequency enhancement module and DCN facilitate the extraction of a broader range of frequency and temporal features.

#### Analysis of EEG data

**Fig 8:**
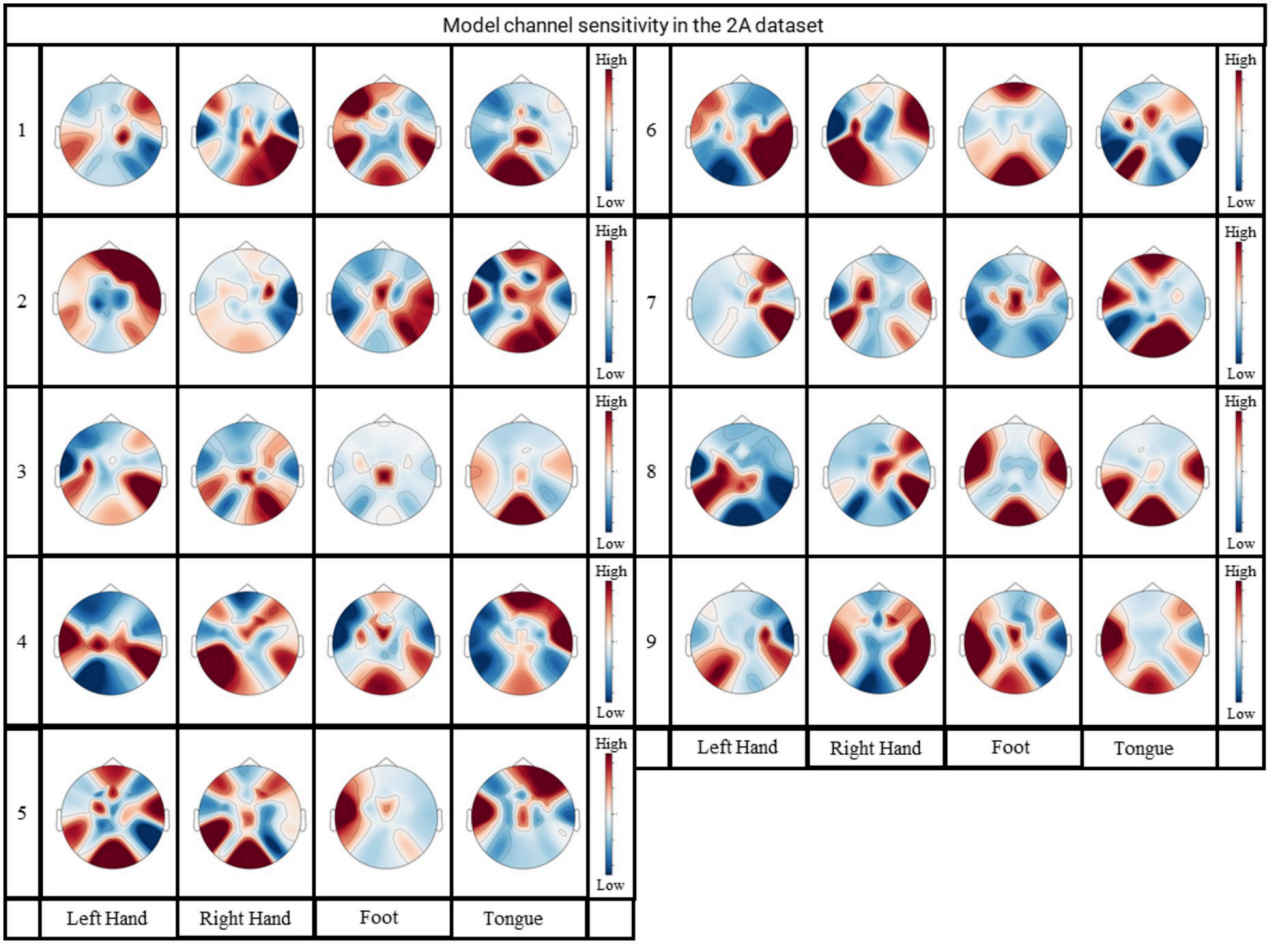
Channel-weighted brain topography of the FDCN-C model in the BCI-C IV 2a dataset.

**Fig 9:**
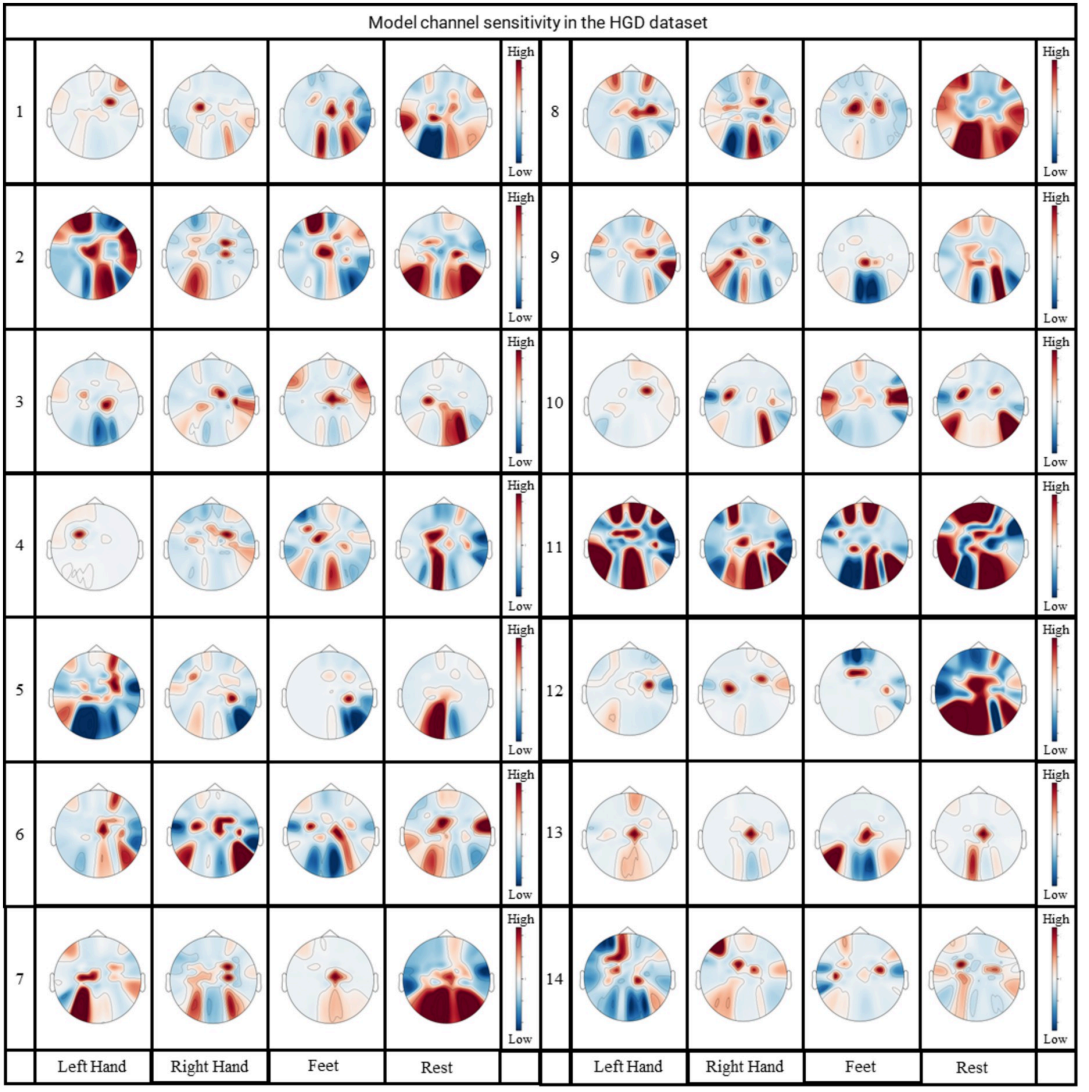
Channel-weighted brain topography of the FDCN-C model in the HGD.

Changing the input data and using a mask that covers some components of the EEG data. By observing the changes of classification accuracy after masking, the weight of the model in different channels can be obtained indirectly. The importance of the EEG data for MI-EEG classification can be analyzed and the sensitivity of this channel for classification can be obtained. This idea is often adopted to verify the interpretability of neural network models [20, 47]. We set the data of one channel to zero and calculate the decrease of the classification accuracy each time. The contribution of each channel for classification can be determined. Figs 8 and 9 display the computed results.

From Figs 8 and 9, the following observations can be made. In the same subject, the topographic maps of channel sensitivities for different MI classes exhibit variations. This confirms that each channel plays a distinct role in the decoding task and exerts an influence on the outcomes. The model’s results are obtained by integrating data from different channels rather than being influenced by noise (which should be consistent across different subjects). Similar to the visual conclusions drawn in the studies of Xie *et al.* [48] and Kwon *et al.* [49], this provides evidence of the model’s accuracy.

Within the same MI class, there is a pronounced distinction in the topographic maps among different subjects, highlighting one of the reasons for the performance degradation of MI-EEG tasks across subjects. This presentation illustrates the differences in EEG data among various subjects, while FDCN-C demonstrates its ability to flexibly extract relevant information from different subjects.

In left-hand MI and right-hand MI tasks, the topographic maps of channel sensitivities exhibit a symmetrical phenomenon, which is more pronounced in the images of subject 6 and subject 8 in the BCI-C IV 2a dataset, as well as in the images of subject 2 and subject 9 in the HGD. Hence, the MI features for left and right hand movements are derived from the contrast between different channels. This indirectly highlights the occurrence of the ERD and ESR phenomena [13], which manifest in different EEG channels. In left-hand MI and right-hand MI, certain channels may exhibit synchronous increases in amplitude and desynchronous decreases in amplitude. Classification models tend to focus on the temporal information associated with the amplitude increases, which can result in symmetric distributions. The visual results of other researchers, as seen in [6, 28], also mention the occurrence of similar phenomena in the channel sensitivity topographic maps for these two MI classes.

Regarding foot MI, a red-colored region appears in the central position of the channel sensitivity topographic maps, which is more pronounced in the images of subject 3 and subject 7 in the BCI-C IV 2a dataset, as well as subject 9 in the HGD. Similar to the experimental results of [13], an occurrence of high channel correlation in the central region was observed. This finding may contributes to further research on more refined paradigms for lower limb MI.

Tongue MI in the the BCI-C IV 2a dataset and Rest MI in the HGD are selected from the regions with darker red blocks among other MI classes.

In summary, the channel-related analysis reveals the relationships between various motor tasks and their corresponding EEG channels. Matching the feature distributions learned by FDCN-C with different motor imagery tasks unveils the underlying relationships between bodily movements and their associated changes in brain activity.

### Discussion

The proposed FDCN-C model is to solve the problem of difficult feature extraction in the MI-EEG classification. The innovating modules include the frequency enhancement module, the deformable convolution network, and the crop classification module. According to two public datasets, the average accuracy of FDCN-C model is higher than that of the baseline CNN models and the hybrid models of CNN and RNN. By hypothesis test, the statistical significance of data from the FDCN-C model and comparison models was demonstrated. Likewise, the ablation study proves the effectiveness of each module in improving classification performance. Based on the T-SNE analysis of its feature extraction ability, the FDCN-C model performs better than the baseline model on feature extraction. Additionally, the working mechanism of the model is examined by altering the input data and indirectly calculating FDCN-C’s attention to various channels.

The DCN modifies the shape and center point position of convolutions in the temporal dimension of EEG data, allowing convolutional kernels to focus on extracting meaningful information more effectively. Compared to the ShallowConvNet-C model without using DCN, the model’s accuracy has improved by 2.83%, providing evidence for the positive impact of DCN on MI-EEG tasks. The frequency enhancement module utilizes convolution at continuous scales to extract information from various frequencies and subsequently retains and integrates the relevant information. Compared to using bandpass filters to obtain frequency bands, this method offers greater flexibility in setting multiple frequency bands and selectively retaining the effective portions. The utilization of skip connections can reduce the training complexity, making the training process more stable. In previous research, skip connections have been demonstrated as essential, providing stability to the training process [36]. In the ablation study, compared to DCN-C, the frequency enhancement module improves the model’s accuracy by 4.29%, demonstrating the effectiveness of frequency domain methods.

However, in terms of the model’s limitations, the first concern is the simplistic treatment of spatial information. In FDCN-C, spatial data extraction is achieved solely through a single set of convolutional kernels. In Figs 8 and 9, the information from multiple EEG channels is indispensable for MI decoding. Therefore, enhancing the extraction of spatial information more effectively becomes crucial for improving the model’s capabilities. In commonly used decoding models such as EEGNET, ShallowNet, and EEGITNet, the discussion on spatial information is relatively lacking. This indicates a potential area for improvement and exploration within the existing models.

The second concern revolves around the introduction of specific structures, resulting in an increase in both the model’s parameter count and computational workload. In the future, approaches like model pruning and knowledge distillation could be employed to optimize the model, addressing its complexity while maintaining performance.

Lastly, the generalizability of the model remains to be tested and validated. This study conducted validation of the model using only two datasets and accomplished subject-independent experiments. In the future, validation will be extended to encompass a broader range of datasets and applied to real-time systems for online verification, further substantiating the model’s efficacy.

Transfer learning and data augmentation have been identified as effective methods for addressing the issue of reduced generalization in cross-subject models. Research in [16, 50] demonstrated the ability of transfer learning and data augmentation to achieve superior EEG decoding models.

## Conclusion

This paper proposed an end-to-end MI-EEG classification model, namely FDCN-C model, which contained a frequency enhancement module, a deformable convolution network, and a crop classification module. In addressing the challenge of extracting frequency information from raw EEG data, we propose a frequency enhancement module. The convolution kernel with continuous time scale was used in the frequency enhancement module to extract different frequency features and then integrate them into the original data. Compared to the commonly used method of extracting frequency information through a bandpass filter bank, the frequency enhancement module demonstrates a more effective capability in capturing information from relevant frequency bands. Then the feature extraction module was used to extract the temporal and spatial domains. A one-dimensional deformable convolution was used in the temporal domain, and the deformable direction and distance of the convolution kernel were controlled by offsets. Compared to fixed-length one-dimensional convolution, the use of variable convolution provides greater flexibility in extracting temporal features from EEG data, resulting in enhanced classification performance. Then the EEG features were integrated by convolution operation in the spatial domain. The crop classification module was used to output the classification results, the expanded convolution was devised to calculate different receptive fields, and the average value was calculated. The proposed model obtained an average accuracy of 80.52% on the BCI-C IV 2a dataset and 96.30% on the HGD, which was higher than state-of-the-art methods. Through visual analysis, the advantageous feature extraction capabilities of the FDCN-C model in MI-EEG classification have been substantiated. Additionally, this paper engages in a comprehensive discussion regarding the EEG topographical features specific to MI-EEG. In the topographic maps of EEG channel correlations during left and right-hand MI, a symmetrical pattern is observed, where EEG channels with high correlation exhibit a left-right symmetry. This phenomenon bears similarity to the ERS and ERD observed in event-related potentials [13]. Simultaneously, it is observed that the correlated channels for foot MI are concentrated in the central region of the brain. We believe that the new modules and experimental findings in this study can assist in the development of more robust brain-computer interface systems.
